# Association between *ALMS 1* variants and early-onset coronary artery disease: a case–control study in Chinese population

**DOI:** 10.1042/BSR20193637

**Published:** 2020-09-01

**Authors:** Shao-Yan Zhang, Chao Xuan, Yi Wang, Shao-Qiang Zhang, Hui Li, Guo-Wei He, Qing-Wu Tian

**Affiliations:** 1 Department of Clinical Laboratory, The Affiliated Hospital of Qingdao University, Qingdao, China; 2 Department of Blood Transfusion, The Affiliated Hospital of Qingdao University, Qingdao, China; 3 Department of Surgery, TEDA International Cardiovascular Hospital, Tianjin and The Affiliated Hospital of Hangzhou Normal University and Zhejiang University, Hangzhou, China; 4 Department of Surgery, Oregon Health and Science University, Portland, Oregon

**Keywords:** ALMS 1, Coronary Artery Disease, Glutamic Acid Repeat, Myocardial Infarction

## Abstract

**Background:** Genome-wide linkage analysis revealed the polymorphism of rs6748040 and glutamic acid repeat are potential pathogenic factors of early-onset myocardial infarction (MI). The present study was designed to investigate the associations of Alström syndrome 1 (*ALMS 1)* gene in Chinese populations with early-onset coronary artery disease (CAD).

**Methods:** The two variants of the *ALMS 1* gene were genotyped in 1252 early-onset CAD patients and 1378 controls using PCR, followed by Sml I restriction enzyme digestion or direct sequencing of the PCR product. The associations were estimated using the odds ratio (OR) and the 95% confidence interval (CI).

**Results:** A significant association between the *ALMS 1* G/A variant and the risk of early-onset MI was detected in G vs.A (OR = 1.371, 95% CI: 1.183–1.589), GG vs. AA (OR = 2.037, 95% CI: 1.408–2.948), dominant genetic model (OR = 1.794, 95% CI: 1.254–2.567), and recessive genetic model (OR = 1.421, 95% CI: 1.177–1.716). 14 glutamic acid repeat (A14) is risk factor for early-onset MI (OR = 1.605, 95% CI: 1.313–1.962) and 17 glutamic acid repeat (A17) is protective factor for the disease (OR = 0.684, 95% CI: 0.601–0.827). These associations were not detected in early-onset CAD patients.

**Conclusions:** Our findings indicated that G/A variant (rs6748040) and glutamic acid repeat polymorphism of the *ALMS 1* gene associated with the risk of early-onset MI in the Chinese population.

## Introduction

Alström syndrome 1 (*ALMS 1)* gene locates on chromosome 2p13. It contains 23 exons spanning 224 kb of the human genome and encodes a 461.2 kDa protein with 4169 amino acids [1]. The ALMS 1 protein is a centrosome and basal body associated protein that contains a large tandem-repeat domain and functions in microtubule organization, particularly in the formation and maintenance of cilia [2,3].

*ALMS 1* widely expressed in almost all tissues that are pathologically affected in patients with Alström Syndrome (AS) [4]. It is a rare and severe autosomal recessive multisystemic disorder characterized by cone-rod dystrophy, hearing loss, obesity, insulin resistance, type 2 diabetes mellitus, dilated cardiomyopathy, and progressive hepatic and renal dysfunction [5,6]. These disorders can also cause serious and even life-threatening problems that affect numerous organ systems including the liver, kidneys, bladder, and lungs. However, the precise molecular mechanisms underlying the multiple organ pathologies have not been fully elucidated.

Two common variants of the *ALMS 1* gene have been linked with the risk of early-onset myocardial infarction (MI) by genome-wide linkage analysis in the Japanese population [7]. The rs6748040 located in the promoter region of *ALMS 1* can affect the gene transcription. Glutamic acid polymorphism in exon 1 could affect the amino acid sequence and function of the protein. According to the principle of genetic diversity, there are differences in genes and phenotypes between different populations due to evolutionary and environmental differences [8,9]. Therefore, it is critical to investigate the associations between the two genetic variations and disease susceptibility in different populations. To our knowledge, there are no studies considering the relationships between common variants of the *ALMS 1* gene and the risk of early-onset coronary artery disease (CAD) in Chinese population. In the present study, we aimed to investigate these associations.

## Materials and methods

In the present study, we followed the methods previously published by our group [10].

### Subjects

In this hospital-based case–control study, all the participants visited The Affiliated Hospital of Qingdao University between January 2013 and February 2019. A total of 1252 patients who met CAD diagnostic criteria were enrolled in the study when their first onset of symptoms and hospitalization for coronary angiography occurred at age ≤50 years. The diagnosis and severity of CAD were assessed by a cardiologist who used angiographic findings. Patients with other serious illnesses were excluded. The 1378 controls were age and sex-matched who did not show any signs or symptoms of cardiovascular events. All patients and controls included in the study signed informed consent before the start of the study. The Ethics Committee of our hospital approved the study (approval number: 20130036), and the protocol was conformed to the ethical guidelines of the Helsinki declaration of 1975.

### Clinical parameters and biochemical measurements

Data on physical examination including smoking and drinking habits, gender, age, height, weight, MI, hypertension, and diabetes mellitus were recorded. According to the National Health and Family Planning Commission, the Chinese Dietary Guidelines (2016), drinking habits are defined as men >25 g/d, women >15 g/d, or a history of excessive drinking within 2 weeks (>80 g/d). We define smokers as those who have smoked continuously or cumulatively for 6 months or more in their lifetime. Whole blood was collected by vacuum blood collection without anticoagulant and was centrifuged at 1500 ***g*** for 15 min. Serum concentrations of low-density lipoprotein cholesterol (LDL-C), triglycerides (TG), high-density lipoprotein cholesterol (HDL-C), total cholesterol (TC), serum creatinine (SCr), fasting blood glucose (FBG), Lipoprotein(a) (Lp(a)), and homocysteine (Hcy) were determined in the morning after fasting of at least 8 h. Serum biochemical indicators were determined using an automatic biochemistry analyzer (Hitachi HCP-7600, Hitachi, Japan) and Series Biochemical Kit (Leadman® Biochemical Co., Ltd, Beijing, China).

### DNA isolation and genotyping

Whole blood was collected by vacuum blood collection using an EDTA-K_2_ anticoagulant. Genomic DNA was isolated by a Blood Genomic DNA Extraction Kit (Tianlong Science and Technology, Xi’an, China) according to the instructions using an NP968 Nucleic Acid Extraction System (Tianlong Science and Technology, Xi’an, China), which was based on a magnetic bead separation method. DNA was extracted from 200 μl whole blood and stored at −80°C. Primers were designed by primer premier software (version 6.0, Palo Alto, CA). The primers for the two common variants of the *ALMS 1* gene were listed in Table 1. The standard PCR protocols for amplifying targets were as follows: one cycle of 5 min at 94°C, then 35 cycles of 30 s at 94°C, 45 s at 52°C/ 55°C, and 45 s at 72°C, followed by 3 min at 72°C using a GeneAmp PCR machine (Tianlong Science and Technology, Xi’an, China), and the size of PCR amplicon was 642 bp. To detect SNP (rs 6748040), PCR products were digested by Sml I and followed by electrophoresis, the size of restriction digestion products was 377 and 265 bp. PCR products were directly sequenced to determine polymorphism of glutamic acid repeat in Genomic Company (Genewiz Biotechnology, Suzhou, China). The reference sequence obtained from NCBI.

**Table 1:** The primers of two polymorphisms in *ALMS1* gene

	Primers
**rs 6748040**	**Forward:** 5′-GGGTCTGCTAAGGTCAAAT-3′
	**Reverse:** 5′-TCTGTAGGTCACTTCTGGTA-3′
**Glutamic acid repeat**	**Forward:** 5′-GAGCGAGACACCAACATGGA-3′
	**Reverse:** 5′-ATACTTTCCAGATGCTGGGGC-3′

### Statistical analysis

An unpaired *t*-test was used to compare continuous variables, and the *χ*^2^ test was used to compare categorical variables. A *Q* test with one degree of freedom was used to test the Hardy–Weinberg equilibrium (*HWE*) [11,12]. In genetic models, the contrast of A vs. G, AA vs. GG, dominant genetic model (AA+AG vs. GG), and recessive genetic model (AA vs. AG+GG) was also investigated. The associations between common variants of the *ALMS 1* gene and the risk of CAD were estimated using the odds ratio (OR) and the 95% confidence interval (CI). Adjusted ORs and 95% CIs after adjustment for age, gender, BMI, hypertension, diabetes, smoking, and biochemical indicators were estimated by logistic regression. Analyses were performed using SPSS software version 11.0, and Stata software version 11.0. *P*<0.05 was considered statistically significant.

## Results

A total of 1252 early-onset CAD patients (mean age 43.88 ± 4.59; 91.70% men) and 1378 controls (mean age 43.63 ± 5.66; 90.85% men) were enrolled in the present study. No significant differences were observed between early-onset CAD patients and controls regarding gender, age, hypertension, and SCr. However, BMI, and levels of FBG, TG, TC, HDL-C, LDL-C, Hcy were significantly elevated in early-onset CAD patients when compared with controls. Besides, the patient group had higher diabetes, and drinking rate compared with controls. In the early-onset CAD patients group, 636 patients were diagnosed with MI. The clinical characteristics of all participants are summarized in Table 2.

**Table 2:** Demographic and clinical characteristics of EOCAD patients and controls

Variable	EOCAD (*n*=1252)	Control (*n*=1378)	*P-*value
**Gender, male *n* (%)** ^#^	1148 (91.70)	1252 (90.85)	0.448
**Age, years** *	43.88 ± 4.59	43.63 ± 5.66	0.216
**BMI (kg/m^2^)** *	26.28 ± 4.35	25.75 ± 5.34	0.006
**Hypertension, *n* (%)** ^#^	353 (28.20)	347 (25.20)	0.081
**Diabetes, *n* (%)** ^#^	234 (18.70)	124 (9.00)	<0.001
**Smoking, *n* (%)** ^#^	517 (41.30)	525 (38.1)	0.094
**Drinking, *n* (%)** ^#^	760 (60.70)	645 (46.80)	<0.001
**FBG, mmol/l** *	5.88 ± 2.12	5.51 ± 1.96	<0.001
**TG, mmol/l** *	1.82 ± 1.73	1.61 ± 1.46	<0.001
**TC, mmol/l** *	4.68 ± 1.43	4.07 ± 1.55	<0.001
**HDL-C, mmol/l** *	2.09 ± 1.26	2.37 ± 1.65	<0.001
**LDL-C, mmol/l** *	2.65 ± 1.28	2.42 ± 1.02	<0.001
**SCr, μmol/l** *	83.63 ± 18.76	83.48 ± 15.63	0.823
**Hcy, μmol/l** *	19.28 ± 6.48	12.77 ± 5.12	< 0.001
**Myocardial infarction, *n* (%)**	636 (50.80)	–	–

Abbreviations: BMI, body mass index; EOCAD, early-onset coronary artery disease; FBG, fasting blood glucose; Hcy, homocysteine; HDL-C, high-density lipoprotein cholesterol; LDL-C, low-density lipoprotein cholesterol; SCr, serum creatinine; TC, total cholesterol; TG, triglyceride.

# Categorical variables are expressed as percentages. The *P*-value of the categorical variables was calculated by χ^2^ test.

* Continuous variables are expressed as mean ± SD. The *P*-value of the continuous variables was calculated by the unpaired *t* test.

**Figure 1:**
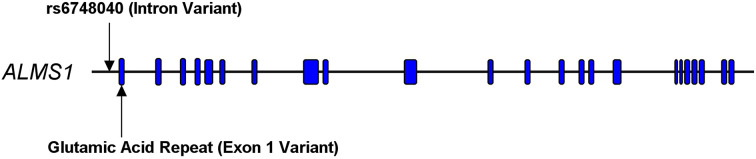
The exon–intron structure of the *ALMS1* gene Exons are shown as black boxes and two variants (rs6748040 and glutamic acid repeat) are indicated by arrows.

**Figure 2:**
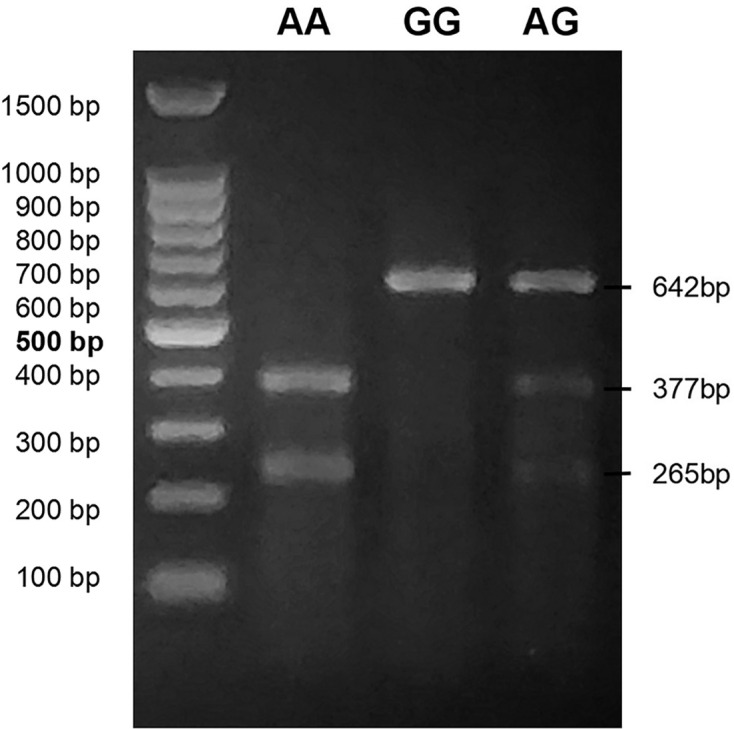
PCR restricted fragment length polymorphism (PCR-RFLP) agarose gel electrophoresis of the *ALMS 1* G/A polymorphism (rs6748040) The 100bp DNA Ladder Marker and restriction digestion products by Sml I were separated in 2% agarose gel. The length of the PCR product that was not digested by Sml I was 642bp, while the length of the PCR products that were completely digested by Sml I was 377bp and 265bp. The PCR products partially digested by Sml I appeared as three fragments (642bp, 377bp, and 265bp).

The exon–intron structure of the *ALMS 1* gene was shown in Figure 1. The G/A variant of the *ALMS 1* gene (rs6748040) was genotyped in all 2630 participants (Table 3). The agarose gel images of PCR and restriction digestion were shown in Figure 2. The distributions of genotype in controls, early-onset CAD, and early-onset MI groups were compatible with *HWE* (*P*_Controls_ = 0.189, *P*_EOCAD_ *=* 0.176, and *P*_EOMI_ *=* 0.696). Significant associations between the *ALMS 1 G*/A variant (rs6748040) and the risk of early-onset MI were detected in G vs. A (OR = 1.371, 95% CI: 1.183–1.589), GG vs. AA (OR = 2.037, 95% CI: 1.408–2.948), dominant genetic model (OR = 1.794, 95% CI: 1.254–2.567), and recessive genetic model (OR = 1.421, 95% CI: 1.177–1.716). After adjusting confounding factors, including age, gender, BMI, hypertension, diabetes, smoking, and biochemical indicators, the allele A was also identified as an independent risk factor for early-onset MI (OR_adjust_ = 1.361, 95% CI: 1.172–1.577, *P*_adjust_ = 4.688 e-5). However, we did not detect any associations between the variant and risk of early-onset CAD. The detailed results are shown in Table 4.

**Table 3:** Genotype frequencies of *ALMS1* gene (rs 6748040) in patient and control groups

Groups	Genotype			*HWE*	Allele	
	GG	GA	AA		G	A
**EOCAD**	711	453	88	0.176	1875	629
**EOMI**	303	275	58	0.696	881	391
**Controls**	777	528	73	0.189	2082	674

Abbreviations: EOCAD, early-onset coronary artery disease; EOMI, early-onset myocardial infarction; *HWE*, Hardy–Weinberg equilibrium.

**Table 4:** The results of *ALMS1* gene polymorphism (rs 6748040) and early-onset MI risk

	*ALMS1* gene polymorphism (rs 6748040)					
	OR (95% CI)	*z*	*P*	OR _adjust_ (95% CI)	*z*	*P*_adjust_
**Early-onset coronary artery disease**						
**G vs. A**	1.036 (0.914–-1.175)	0.56	0.577	1.028 (0.903–1.162)	0.43	0.668
**GG vs. AA**	1.317 (0.950–1.826)	1.65	0.098	1.306 (0.938–1.816)	1.58	0.113
**Dominant genetic model**	1.352 (0.981–1.862)	1.84	0.065	1.336 (0.973–1.846)	1.77	0.076
**Recessive genetic model**	0.984 (0.843–1.148)	0.21	0.835	0.991 (0.852–1.158)	0.12	0.908
**Early-onset myocardial infarction**						
**G vs. A**	1.371 (1.183–1.589)	4.19	**2.790 e-5**	1.361 (1.172–1.577)	4.07	**4.688 e-5**
**GG vs. AA**	2.037 (1.408–2.948)	3.78	**1.568 e-4**	2.024 (1.394–2.935)	3.71	**2.054 e-4**
**Dominant genetic model**	1.794 (1.254–2.567)	3.20	**1.000 e-3**	1.780 (1.241–2.555)	3.13	**1.748 e-3**
**Recessive genetic model**	1.421 (1.177–1.716)	3.65	**2.622 e-4**	1.406 (1.160–1.702)	3.48	**4.939 e-4**

Abbreviations: CI, confidence interval; OR, odds ratio.

Adjust confounding factors including age, gender, BMI, hypertension, diabetes, smoking, and biochemical indicators.

**Figure 3:**
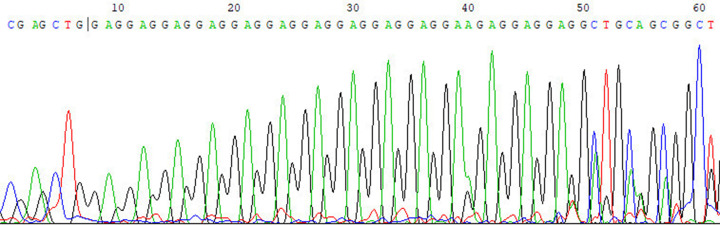
The typical sequence chromatogram of glutamic acid repeats (A14) in exon 1 of *ALMS 1* gene

We detected glutamic acid repeats (13–22 amino acid) consisting of a common structure of (GAG)_n_GAA(GAG)_3._ A14 (26.49% in controls) and A17 (49.78% in controls) were the frequent alleles in the glutamic acid repeats of the *ALMS 1* gene (Table 5). The typical image of the sequencing result was shown in Figure 3. The distributions of glutamic acid repeat in all participants were list in Table 4. The allele frequency for A14 was increased in patients with early-onset MI compared with controls (OR = 1.605, 95% CI: 1.313–1.962). After adjusting confounding factors, including age, gender, BMI, hypertension, diabetes, smoking, and biochemical indicators, the association remains (OR_adjust_ = 1.599, 95% CI: 1.306–1.953). On the contrary, A17 allele frequency was decreased in early-onset MI patients, it was considered as a protective factor (OR = 0.684, 95% CI: 0.565–0.827). The results are consistent after logistic regression analysis (OR_adjust_ = 0.687, 95% CI: 0.570–0.832). The detailed results are shown in Table 6.

**Table 5:** Glutamic acid repeat variant frequency of of *ALMS1* gene in patients and control groups

Alleles	Frequency		
	EOCAD (%)	EOMI (%)	Controls (%)
**A13**	3 (0.24%)	1 (0.16)	2 (0.15)
**A14**	369 (29.47)	233 (36.64)	365 (26.49)
**A15**	29 (2.32)	13 (2.04)	39 (2.83)
**A16**	95 (7.59)	57 (8.96)	91 (6.60)
**A17**	594 (47.44)	257 (40.41)	686 (49.78)
**A18**	96 (7.67)	45 (7.08)	112 (8.13)
**A19**	24 (1.92)	12 (1.89)	27 (1.96)
**A20**	40 (3.19)	18 (2.83)	52 (3.77)
**A21**	2 (0.16%)	—	3 (0.22)
**A22**	—	—	1 (0.07)
Short*	496	304	497

Abbreviations: EOCAD, early-onset coronary artery disease; EOMI, early-onset myocardial infarction.

**Table 6:** The results of glutamic acid repeat variant and early-onset coronary artery disease risk

	Early-onset coronary artery disease						Early-onset myocardial infarction					
	OR (95% CI)	*z*	*P*	OR _adjust_ (95% CI)	*z*	*P*_adjust_	OR (95% CI)	*z*	*P*	OR _adjust_ (95% CI)	*z*	*P*_adjust_
**A14**	1.160 (0.978–1.375)	1.70	0.088	1.153 (0.970–1.366)	1.63	0.103	1.605 (1.313–1.962)	4.61	4.027 e-6	1.599 (1.306–1.953)	4.57	4.819 e-6
**A15**	0.814 (0.500–1.325)	0.83	0.408	0.821 (0.508–1.334)	0.80	0.423	0.716 (0.380–1.352)	1.03	0.303	0.724 (0.388–1.361)	1.01	0.313
**A16**	1.161 (0.862–1.565)	0.98	0.326	1.152 (0.852–1.554)	0.92	0.356	1.392 (0.986–1.966)	1.88	0.060	1.384 (0.976–1.955)	1.83	0.067
**A17**	0.911 (0.781–1.061)	1.20	0.231	0.920 (0.791–1.070)	1.08	0.279	0.684 (0.565–0.827)	3.91	9.230 e-5	0.687 (0.570–0.832)	3.89	9.973e-5
**A18**	0.939 (0.707–1.247)	0.44	0.662	0.949 (0.716–1.258)	0.36	0.716	0.861 (0.601–1.233)	0.82	0.413	0.870 (0.612–1.241)	0.77	0.440
**A19**	0.978 (0.561–1.704)	0.08	0.937	0.986 (0.571–1.715)	0.05	0.960	0.962 (0.484–1.912)	0.11	0.913	0.971 (0.492–1.922)	0.08	0.933
**A20**	0.842 (0.553–1.280)	0.81	0.420	0.849 (0.561–1.290)	0.77	0.440	0.743 (0.431–1.280)	1.07	0.284	0.749 (0.439–1.288)	1.05	0.293
**Short** *	1.163 (0.993–1.362)	1.87	0.061	1.152 (0.980–1.351)	1.73	0.084	1.623 (1.342–1.964)	4.98	6.358 e-7	1.618 (1.335–1.955)	4.94	7.619 e-7

Abbreviations: CI, confidence interval; OR, odds ratio.

Adjust confounding factors including age, gender, BMI, hypertension, diabetes, smoking, and biochemical indicators.

* Alleles were 9–16 glutamic acid repeat.

## Discussion

This is the first large population-based case–control study investigating common variations of the *ALMS 1* gene and early-onset CAD in the Chinese population. In the present study, we demonstrated that the G/A variant (rs6748040) of the *ALMS 1* promoter region and glutamic acid repeat polymorphism of exon 1 was closely related to the risk of early-onset MI in the Chinese population. However, we found no evidence of associations between these two variations of the *ALMS 1* gene and early-onset CAD.

*ALMS 1* gene located on chromosome 2p13.1, encoding a protein of 4169 amino acids. The ALMS 1 protein is widely expressed in various tissues of human beings, but its function is still unknown. Fibroblasts with *ALMS 1* mutation continue to proliferate and secrete high levels of the extracellular matrix. This may be an important pathological mechanism for the formation of fibrous plaque in the coronary arteries and the development of atherosclerosis, ultimately leading to CAD, especially MI. It is already clear that *ALMS 1* gene mutations are closely related to AS and is the pathogenic gene of the disease. Although functional studies of *ALMS 1* may provide insight into how variants of this gene to produce its pathological effects, the function of the gene has not been fully elucidated. This gene encodes an ALMS 1 protein which is a component of the centrosome [13]. Recent studies suggested that it is involved in microtubule organization and intracellular transport [14,15]. AS is a multi-system disease, mainly characterized by rod-cone retinal degeneration and dilated cardiomyopathy with heart failure in infancy [16]. AS also produces many metabolic abnormalities, such as adolescent obesity with hypertriglyceridemia, liver steatosis, insulin resistance, and type 2 diabetes mellitus [17–19]. It would be expected that AS patients could develop early-onset CAD. Coincidentally, the *ALMS 1* gene was identified as a genetic risk marker for early-onset MI by genome-wide linkage analysis in the Japanese population in 2013 [7]. Genome-wide affected sib-pair linkage study in 221 Japanese families with early-onset CAD linked the chromosome 2p13 and early-onset MI. The *ALMS 1* gene is the candidate gene within the linkage region. Subsequent analysis in 2186 Japanese patients with MI and 6026 controls proved that a strong association between rs6748040 and early-onset MI. Besides, glutamic acid repeat polymorphism in exon 1 of the *ALMS 1* gene has also been proved to be an important risk factor for early-onset MI.

Worldwide, CAD remains the leading cause of death and disability. Early-onset CAD affects young and middle-aged individuals and is more harmful than conventional CAD. There is no clear age range for early-onset CAD, but many studies have limited it to under 50 years of age. As a special type of CAD, early-onset CAD has particular components of etiology, including family heredity, lipid metabolism, gender composition, and other risk factors. In our previous studies, we demonstrated that asymmetric dimethylarginine, uric acid, and homocysteine associated with the presence and severity of early-onset CAD [10,20,21]. Familial aggregation strongly indicated the presence of genetic factors for increased susceptibility to the disease. Our findings also indicated that abnormal lipid metabolism and genetic factors may play more important roles in the pathogenesis of early-onset CAD [22,23]. To our knowledge, no study has been described on *ALMS 1* gene variants (rs6748040 and glutamic acid repeat polymorphism) and early-onset CAD risk in the Chinese population. According to the principle of genetic diversity, differences in genes and phenotypes between races result from differences in evolution and environment. Therefore, it is critical to study these associations in the Chinese population.

In the present study, we evaluated the associations between the two variants of the *ALMS 1* gene (rs6748040 and glutamic acid repeat polymorphism) and the risk of early-onset CAD in 1252 early-onset CAD patients and 1378 controls. We confirmed that the *ALMS1* G/A variant (rs6748040) was significantly related to an increased risk of early-onset MI, and individuals carrying allele G have a significantly increased risk of the disease (OR_adjust_ = 1.361, 95% CI: 1.172–1.577; *P* = 4.688 e-5). In addition, 14 glutamic acid repeat (A14) is risk factor for early-onset MI (OR_adjust_ = 1.599, 95% CI: 1.306–1.953; *P*=4.819 e-6). On the contrary, 17 glutamic acid repeat (A17) is protective factor for the disease (OR_adjust_ = 0.687, 95% CI: 0.570–0.832; *P*=9.973 e-5). No associations were detected between the two variants and the risk of early-onset CAD.

Our study has some limitations. First, although we selected gender- and age-matched individuals without signs or symptoms of CAD as the control group, it should be noted that the controls did not undergo angiography. Second, geographic variations in the prevalence of *ALMS 1* variants in the Chinese population may bias the results of the single-center case–control studies. Third, there is always a controversy about which confounding factors should be used for multivariate analysis. One suggested that all differential confounding factors should be used for correction. The other is that all confounding factors should be used for correction. In our previous studies, we tended to use the second approach. In fact, no matter which method is used, the results will basically not change significantly.

In conclusion, we observed that the two variants of the *ALMS 1* gene were significantly associated with the risk of early-onset MI. However, the specific molecular pathological mechanism needs to be clarified in the following studies.

## Data Availability

The data sets used and/or analyzed during the current study are available from the corresponding author on reasonable request.

## Abbreviations

*ALMS 1*: Alström syndrome 1 (gene)
BMI: body mass index
CAD: coronary artery disease
CI: confidence interval
HWE: Hardy–Weinberg equilibrium
MI: myocardial infarction
OR: odds ratio
